# A prospective six-month audit of inpatient hypoglycemia in step-down general medical and geriatric wards

**DOI:** 10.7150/ijms.63381

**Published:** 2021-10-03

**Authors:** Penny Dwyer, Jocelyn J Drinkwater, P Gerry Fegan, Wendy A Davis, Timothy M E Davis

**Affiliations:** 1 Medical School, University of Western Australia, Fremantle Hospital, Fremantle, Western Australia, Australia.; 2 Department of Endocrinology and Diabetes, Fiona Stanley Hospital, Perth, Western Australia, Australia.

## Abstract

This study aimed to assess the incidence and associates of hypoglycemia in patients transferred after stabilization on an Acute Medical Unit to two general medical or two geriatric wards at an urban Australian hospital. In a six-month audit representing 20,284 patient-days of observation, 59 inpatients experienced hypoglycaemia (blood glucose ≤3.9 mmol/L) during 65 hospitalizations. Inpatients experiencing hypoglycemia accounted for 7.2% of all inpatient bed-days, a figure that was greater for general medical (9.2% of bed-days) compared with geriatric (6.0% of bed-days) wards (*P*<0.001). Inpatient hypoglycemia often had no precipitant such as a missed/delayed meal, occurred disproportionately at night (41% of episodes), was severe (blood glucose ≤3.0 mmol/L) in one-third of cases, and appeared more frequent in patients with psychiatric/cognitive issues. These data highlight the ongoing issue of hypoglycemia in relatively stable inpatients in an era of blood glucose-lowering therapies associated with a low rate of this acute metabolic complication.

## Introduction

Hypoglycemia complicating inpatient diabetes management has been associated with increased morbidity, length of stay, cost and mortality 1-3. Reports of its frequency have shown variable results, reflecting heterogeneity in patient characteristics, blood glucose thresholds and methods of glycemic monitoring. In studies of non-critically ill patients in US general medical wards, between 3.5% 4 and 10.1% 5 had at least one mixed capillary blood glucose level (BGL) ≤3.9 mmol/L during hospitalization. Contemporary Australian data using the same biochemical threshold appear similar. Although utilizing a denominator of inpatients with known or newly-detected diabetes, the 16.0% 6 and 29.9% 7 of this sub-group experiencing hypoglycemia equates to between 3.8% and 7.0% of all inpatients assuming that approaching 1 in 4 of hospitalized Australians have diabetes 7,8.

Although exclusion criteria such as critical illness ensure that inpatients at abnormally high risk do not bias estimates, available data still include people admitted because of hypoglycemia or with acute non-critical conditions associated with glycemic instability 4-7. The burden of hypoglycemia in stabilized patients may differ from that in the general inpatient population, especially since the introduction of the Acute Medical Unit (AMU) has improved short-term outcomes including early discharge 9. In addition, elderly inpatients experiencing hypoglycemia are at increased risk of hypoglycemia^3^ and its sequelae such as falls 10 and death 11. Nevertheless, there are few published studies in the elderly. The aim of the present study was, therefore, to audit the incidence and associates of hypoglycemia in patients transferred from an AMU for ongoing management on general medical or geriatric wards at an urban Australian hospital.

## Materials and Methods

Approval was obtained from the Southern Metropolitan Health Service Human Research Ethics Committee to audit hypoglycemic events in the two general medical and two geriatric wards at Fremantle Hospital (FH) over a six-month period (RGS0000000001). FH Inpatients are transferred after initial stabilization in the neighbouring Fiona Stanley Hospital AMU. A Study Nurse visited the four FH wards daily to document all hypoglycemia (mixed capillary BGL ≤3.9 mmol/L) recorded in medical/nursing notes and blood glucose monitoring charts over the previous 24 hours, with ascertainment of weekend events every Monday morning. Other relevant data were collected, including precipitating factors, symptoms, therapies for diabetes/other illnesses, co-morbidities, hypoglycemia-related complications, and length of stay.

Hypoglycemia was categorized as Level 1 (BGL ≤3.9 mmol/L, the neuroendocrine response threshold) or Level 2 (BGL ≤3.0 mmol/L, the threshold for neuroglycopenic symptoms requiring immediate action) 12. We did not classify Level 3 episodes (altered mental/physical functioning requiring second party assistance) 12 since inpatients typically receive nursing care for any hypoglycemia and may be unable to self-treat because of unrelated mental/physical dysfunction. Total daily inpatient bed-day counts for the four wards audited were collated for the observation period.

## Results

During 20,284 patient-days (38.1% on the general medical, 61.9% on the geriatric wards), 59 inpatients with hypoglycemia were identified from 65 hospitalizations. On admission (the first occasion if there were subsequent hospitalizations) they were of mean±SD age 73.1±12.5 (range 44.1-96.7) years, 34 (57.6%) were males, and 51 (86.4%) had type 2 diabetes. Diabetes duration, available for 35 patients, was a median [inter-quartile range (IQR)] 20 [15-28] years. The majority of the 59 patients (78.9%) were insulin-treated, and 12.5% were taking a sulfonylurea with one of these also using insulin. Five (8.5%) were taking a dipeptidyl peptidase-4 inhibitor, but none were treated with a sodium-glucose cotransporter 2 inhibitor or glucagon-like peptide 1 receptor agonist.

Some patients were transferred between wards and/or were admitted to a different ward during subsequent hospitalization and this has been accommodated in data analysis. A total of 173 hypoglycemic BGLs were captured (median [IQR] 3.3 [2.9-3.7] mmol/L). Of these, 119 (68.8%) were a first or a new index episode, 11 (6.4%) occurred up to 60 minutes after the index event and were classified as the same event, and 43 (24.9%) occurred between one and 24 hours after the index episode and were considered recurrences 13. Sixty-five index episodes (54.6%) occurred on general medical wards and 54 (45.4%) on geriatric wards. There were between one and eight index episodes per patient but over half the patients (n=36; 61.0%) experienced only one episode.

Of the total number of events, 39 (22.5%) had documented typical autonomic warning symptoms, 49 (41.2%) occurred between 2200 and 0600 hours, and 35 (29.4%) were Level 2. Seventeen (26.2%) of the episodes on general medical wards were Level 2 versus 18 (33.3%) on geriatric wards (*P*=0.392). Most episodes (92.4%) did not have a clear precipitating event such as delayed/missed meal or blood glucose-lowering medication administration error. However, 23 (39.0%) of the 59 patients had a psychiatric illness, a history of drug/alcohol misuse, and/or dementia, cognitive impairment or an intellectual disability. In addition, 33 (55.9%) were nursing home or hostel residents, were awaiting placement, or had a carer or home care support services.

Inpatients experiencing hypoglycemia accounted for 1,467 bed-days (or 7.2% of total bed-days, with an average length of stay of 23 days) over the six months, comprising 710 on general medical wards (9.2% of all general medical bed-days) and 757 on geriatric wards (6.0% of all geriatric bed-days) (*P*<0.001). Of the 59 patients with hypoglycemia, two (3.4%) died in hospital, three (5.1%) within 30 days of discharge, and 11 (18.6%) between one and 12 months post-discharge. The 16 (27.1%) who died within one year of first hospitalization were significantly older at first admission than those who did not (81.0±9.0 vs 70.1±12.4 years, *P*=0.002), but there was no sex difference (*P*=0.241).

A comparison of the hypoglycemic patients by location at first admission is summarized in Table 1. General medical patients were younger and less likely to be of Anglo-Celt ethnicity, but there were no other statistically significant demographic or diabetes-related differences. Mortality was also similar. Two (8.7%) general medical versus no geriatric patients died in hospital (*P*=0.148), and 5 (21.7%) versus 11 (30.6%) patients, respectively, had died 12 months post-admission (*P*=0.556).

## Discussion

The present data show that hypoglycemia remains relatively frequent in patients on step-down general medical/geriatric wards. Although conditions associated with a marked increase in hypoglycemia risk were excluded due to prior AMU stabilization, the patients with documented hypoglycemia accounted for 7.2% of all bed-days on the audited wards. This is greater than the estimated equivalent figure of 3.8% to 7.0% for all Australian inpatients with at least one documented BGL ≤3.9 mmol/L 6-8. Most of the present patients experienced only a single episode, but a disproportionate number of events occurred overnight, most were not associated with typical symptoms, and approaching one third were Level 2. Patients with hypoglycemia contributed less to total bed days on the geriatric compared with the general medical wards.

Because of their clinical complexity, general medical inpatients in the present audit were likely to have been older than in previous Australian studies 6,7. Their relatively high hypoglycemia rate could reflect this since age is a recognized risk factor for inpatient hypoglycemia 3. However, this hypothesis seems at odds with the fact that inpatients on the geriatric wards were significantly less likely to experience hypoglycemia than their younger general medical counterparts. Nevertheless, most recent management guidelines 14, including those from Australia 15, recommend treatment de-intensification to reduce the hypoglycemia risk and avoid polypharmacy in older individuals with diabetes. This may have explained the lower rate on the geriatric wards, but a lower acuity of illness amongst the older patients may have also contributed.

Hypoglycemia was 40% more likely to occur overnight than during the rest of the 24-hour period. An increased frequency of nocturnal versus daytime hypoglycemia has also been found in studies utilizing continuous subcutaneous glucose monitoring (CSGM) in outpatients with type 2 diabetes 16. Contrary to the results of studies in other countries 17, we did not find that delayed or missed meals was a major contribution to inpatient hypoglycemia. This may have reflected the close supervision of meals and medications in the well-resourced Australian ward environment. Our data do, however, suggest that vulnerable patients, including those with psychiatric and cognitive issues, are over-represented. A more detailed analysis of these data was beyond the scope of the present study. About one third of the hypoglycemic episodes detected were Level 2 regardless of location. This finding is consistent with other Australian 6 and US 18 data. Although severe hypoglycemia is a recognised risk factor for increased mortality both during and after admission 19, there were few inpatient deaths in the present study and those within the 12 months post-discharge were consistent with the age of the sample and frequent multimorbidity.

A limitation of the present study, as with all studies utilizing mixed capillary BGL data, is that there may have been hypoglycemic episodes that were missed because they were asymptomatic and occurred outside the fixed times for BGL measurement. Indeed, only the minority of captured episodes were symptomatic which is consistent with increased hypoglycemia unawareness in older patients 20 but which could also reflect masking by factors such as coincident pain or cognitive impairment. In any case, there are reports of a relatively high rate of undetected hypoglycemia revealed by CSGM in general outpatients with diabetes 16. The specified times for BGL testing were typically before each meal and at bedtime, but not overnight, in those on insulin, but the schedule could be personalized by the clinical team and was usually less intensive in non-insulin-treated patients. Some collateral data such as the timing of hypoglycemia relative to meals was not consistently recorded in the medical/nursing notes. The strengths of the study are its prospective design, defined population, and relatively long observation period.

In conclusion, the present data highlight the ongoing issue of hypoglycemia in clinically relatively stable inpatients. Those who experienced hypoglycaemia had longer duration diabetes and were thus mostly treated with insulin rather than the newer oral or injectable blood glucose-lowering therapies that are associated with a low rate of this acute metabolic complication 21. The present data suggest that vulnerable patient sub-groups such as those with psychiatric and/or cognitive issues may be at particular risk. A significant proportion of the hypoglycemic episodes, approximately one third, were below the threshold for neuroglycopenic symptoms and thus indicated the need for immediate action 12, while many occurred overnight. These latter observations provide an argument for routine utilization of relatively non-invasive CSGM, perhaps even with telemetry 22, in insulin-treated and other at-risk inpatients so that imminent hypoglycemia can be identified and circumvented.

## Figures and Tables

**Table 1:** Characteristics of inpatients at first admission to Fremantle Hospital when hypoglycaemia was recorded by ward first admitted to (medical versus geriatric)

	General medical ward	Geriatric ward	*P*-value
N (%)	36 (61.0)	23 (39.0)	
Age (years)	69.4±12.9	78.8±9.5	0.004
Sex (% male)	55.6	60.9	0.790
Anglo-Celt ethnicity (%)	66.7	100	0.002
Non-English speaking (%)	8.3	0	0.274
Type 2 diabetes (%)	83.3	91.3	0.464
Diabetes duration (years)*	20.0 [11.5-27.5]	22.0 [16.0-29.0]	0.369
On insulin (%)	82.4	73.9	0.517
On sulphonylureas (%)	14.7	9.1	0.692
Smoking status (%)**			
Never	31.8	23.5	
Ex-	40.9	70.6	0.156
Current	27.3	5.9	
Consumes alcohol (%)***	36.0	23.5	0.505
Mental health problems, dementia, or alcohol/drug misuse (%)	22.2	8.7	0.288

Data are presented as percentages, mean ± standard deviation, median [interquartile range]. Missing data *n=24, **n=20, ***n=17.

## References

[1] Brutsaert E, Carey M, Zonszein J (2014). The clinical impact of inpatient hypoglycemia. J Diabetes Complications.

[2] Cruz P (2020). Inpatient hypoglycemia: the challenge remains. J Diabetes Sci Technol.

[3] Hulkower RD, Pollack RM, Zonszein J (2014). Understanding hypoglycemia in hospitalized patients. Diabetes Manag (Lond).

[4] Cook CB, Kongable GL, Potter DJ, Abad VJ, Leija DE, Anderson M (2009). Inpatient glucose control: a glycemic survey of 126 U.S. hospitals. J Hosp Med.

[5] Boucai L, Southern WN, Zonszein J (2011). Hypoglycemia-associated mortality is not drug-associated but linked to comorbidities. Am J Med.

[6] Kyi M, Colman PG, Rowan LM, Marley KA, Wraight PR, Fourlanos S (2019). Glucometric benchmarking in an Australian hospital enabled by networked glucose meter technology. Med J Aust.

[7] Malabu UH, Adegboye O, Hayes OG (2020). Influence of Ethnicity on outcomes of diabetes inpatient hypoglycemia: an Australian perspective. J Endocr Soc.

[8] Lan NSR, Li C, Fegan PG (2019). Diabetes prevalence is high in hospital patients: a Western Australia perspective. Intern Med J.

[9] Suthers B, Pickles R, Boyle M, Nair K, Cook J, Attia J (2012). The effect of context on performance of an acute medical unit: experience from an Australian tertiary hospital. Aust Health Rev.

[10] Nelson JM, Dufraux K, Cook PF (2007). The relationship between glycemic control and falls in older adults. J Am Geriatr Soc.

[11] Kagansky N, Levy S, Rimon E (2003). Hypoglycemia as a predictor of mortality in hospitalized elderly patients. Arch Intern Med.

[12] American Diabetes Association (2021). 6. Glycemic targets: standards of medical care in diabetes -2021. Diabetes Care.

[13] Ulmer BJ, Kara A, Mariash CN (2015). Temporal occurrences and recurrence patterns of hypoglycemia during hospitalization. Endocr Pract.

[14] American Diabetes Association (2021). 12. Older adults: standards of medical care in diabetes - 2021. Diabetes Care.

[15] The Royal Australian College of General Practitioners (2020). Management of type 2 diabetes in older people and residential aged care facilities M. East Melbourne, Vic: RACGP, 2020. Management of type 2 diabetes: A handbook for general practice. Melbourne: RACGP.

[16] Weber KK, Lohmann T, Busch K, Donati-Hirsch I, Riel R (2007). High frequency of unrecognized hypoglycaemias in patients with Type 2 diabetes is discovered by continuous glucose monitoring. Exp Clin Endocrinol Diabetes.

[17] Pratiwi C, Mokoagow MI, Made Kshanti IA, Soewondo P (2020). The risk factors of inpatient hypoglycemia: A systematic review. Heliyon.

[18] Varghese P, Gleason V, Sorokin R, Senholzi C, Jabbour S, Gottlieb JE (2007). Hypoglycemia in hospitalized patients treated with antihyperglycemic agents. J Hosp Med.

[19] Turchin A, Matheny ME, Shubina M, Scanlon JV, Greenwood B, Pendergrass ML (2009). Hypoglycemia and clinical outcomes in patients with diabetes hospitalized in the general ward. Diabetes Care.

[20] Bremer JP, Jauch-Chara K, Hallschmid M, Schmid S, Schultes B (2009). Hypoglycemia unawareness in older compared with middle-aged patients with type 2 diabetes. Diabetes Care.

[21] Freeman J (2019). Management of hypoglycemia in older adults with type 2 diabetes. Postgrad Med.

[22] Spanakis EK, Levitt DL, Siddiqui T (2018). The effect of continuous glucose monitoring in preventing inpatient hypoglycemia in general wards: the glucose telemetry system. J Diabetes Sci Technol.

